# Mr. Goldson, in Reply to Dr. Walker

**Published:** 1804-07-01

**Authors:** W. Goldson

**Affiliations:** Portsea


### *To the Editors of the Medical and Phyſical Journal.*

Gentlemen,

THE Pamphlet, which is the subject of the Remarks at p. 505 of your last Journal, contains cases of small-pox at the distance of *three years* from vaccination, happening in subjects, who, during the intermediate space, had been submitted to the strongest influence of variolous infection. It is addressed to the Directors of the Vaccine Institution, requesting them, in terms I believe perfectly respectful, and with that degree of moderation which becomes the subject, to turn their attention to it, and seriously to investigate, what I am forcibly led to conceive a defect, hitherto not *publicly* noticed in Vaccination. Prior to its publication, I sent a letter addressed to the Secretary, at No. 14, Salisbury Square, accompanied with a copy of the publication, requesting that it might be delivered to the Directors in my name, as a mark of my attention. I presume the letter was delivered, although I never received any answer to it. But Dr. Walker, who is the author of the Remarks I allude to, can inform you, as he resides, I believe, at the house where the letter was left.

In those Remarks, although on a subject so important to the interests of society, he has been pleased, at first sight, to deem them cases of spurious cow-pox, without adverting to their resistance of small-pox, so evidently during a limited period; a feature so prominent as to distinguish them from any *hitherto* brought before the public. These remarks are likewise conveyed in language so illiberal, and are replete with so many misrepresentations, which must be deemed wilful, that I should have suffered them to have passed unnoticed, had not the author been principal Vaccinator, under the Directors of an Institution, to whom the Pamphlet was respectfully addressed. From their characters however as gentlemen, I am confident, it was sent to your Journal without their knowledge.

A postscript is added to the Remarks, asserting, that, "Dr. Waller of Portsmouth had just made an application at the Central House of the Royal Jennerian Society, for vaccine matter, with the remark, that that which they have there cannot be depended on; and on that account he sends to the Society, where he reckons upon being furnished with the genuine."

If Dr. Waller ever made such an application, it must be deemed extremely illiberal, not to me alone, but to the whole of the profession in the vicinity. This, however, he publicly denies, and says, he never had any correspondence with Dr. Walker, and never gave any authority for such an assertion.

To men of *real* humanity, and to lovers of the truth, I am confident the importance of the subject will be an inducement to investigate it. With respect to them, I need not, but with regard to Dr. Walker, I must request you will insert the concluding sentence of the pamphlet:

"It is far from my wish to provoke controversy; I only ask for further investigation. Vaccine Inoculation must stand by its own merits, or fall from its own defects. To suffer zeal for the discovery to shut their eyes to conviction, and, by deeming every failure spurious, to conceal it, is beneath the dignity of the profession. If it does not ultimately prove a permanent prophylactic, the consequence must be, that the small-pox, at some future period, will become a greater scourge to the world than ever, independent of the distress of mens' minds from their being left in such a dreadful state of anxiety. Let me beg therefore, that in conducting this investigation, the words of Dr. Jenner may be had in remembrance. "I again repeat my earnest hope that it may be conducted with that calmness and moderation which should ever accompany a philosophical research."

I am, &c.

W. GOLDSON.

